# Co-cultures of colon cancer cells and cancer-associated fibroblasts recapitulate the aggressive features of mesenchymal-like colon cancer

**DOI:** 10.3389/fimmu.2023.1053920

**Published:** 2023-05-16

**Authors:** Esther Strating, Mathijs P. Verhagen, Emerens Wensink, Ester Dünnebach, Liza Wijler, Itziar Aranguren, Alberto Sanchez De la Cruz, Niek A. Peters, Joris H. Hageman, Mirjam M. C. van der Net, Susanne van Schelven, Jamila Laoukili, Riccardo Fodde, Jeanine Roodhart, Stefan Nierkens, Hugo Snippert, Martijn Gloerich, Inne Borel Rinkes, Sjoerd G. Elias, Onno Kranenburg

**Affiliations:** ^1^ Laboratory Translational Oncology, Division of Imaging and Cancer, University Medical Center Utrecht, Utrecht, Netherlands; ^2^ Department of Pathology, Erasmus Medical Center, Rotterdam, Netherlands; ^3^ Department of Medical Oncology, Division of Imaging and Cancer, University Medical Center Utrecht, Utrecht, Netherlands; ^4^ Center for Translational Immunology, University Medical Center Utrecht, Utrecht University, Utrecht, Netherlands; ^5^ Princess Máxima Center for Pediatric Oncology, Utrecht, Netherlands; ^6^ Center for Molecular Medicine, Division LAB, University Medical Center Utrecht, Utrecht, Netherlands; ^7^ Department of Epidemiology, Julius Center for Health Sciences and Primary Care, University Medical Center Utrecht, Utrecht, Netherlands; ^8^ Utrecht Platform for Organoid Technology, Utrecht University, Utrecht, Netherlands

**Keywords:** colorectal cancer, CMS4, immunosuppressive, microenvironment, cancer-associated fibroblast (CAF)

## Abstract

**Background:**

Poor prognosis in colon cancer is associated with a high content of cancer-associated fibroblasts (CAFs) and an immunosuppressive tumor microenvironment. The relationship between these two features is incompletely understood. Here, we aimed to generate a model system for studying the interaction between cancer cells and CAFs and their effect on immune-related cytokines and T cell proliferation.

**Methods:**

CAFs were isolated from colon cancer liver metastases and were immortalized to prolong lifespan and improve robustness and reproducibility. Established medium and matrix compositions that support the growth of patient-derived organoids were adapted to also support CAF growth. Changes in growth pattern and cellular re-organization were assessed by confocal microscopy, live cell imaging, and immunofluorescence. Single cell RNA sequencing was used to study CAF/organoid co-culture-induced phenotypic changes in both cell types. Conditioned media were used to quantify the production of immunosuppressive factors and to assess their effect on T cell proliferation.

**Results:**

We developed a co-culture system in which colon cancer organoids and CAFs spontaneously organize into superstructures with a high capacity to contract and stiffen the extracellular matrix (ECM). CAF-produced collagen IV provided a basement membrane supporting cancer cell organization into glandular structures, reminiscent of human cancer histology. Single cell RNA sequencing analysis showed that CAFs induced a partial epithelial-to-mesenchymal-transition in a subpopulation of cancer cells, similar to what is observed in the mesenchymal-like consensus molecular subtype 4 (CMS4) colon cancer. CAFs in co-culture were characterized by high expression of ECM components, ECM-remodeling enzymes, glycolysis, hypoxia, and genes involved in immunosuppression. An expression signature derived from CAFs in co-culture identified a subpopulation of glycolytic myofibroblasts specifically residing in CMS1 and CMS4 colon cancer. Medium conditioned by co-cultures contained high levels of the immunosuppressive factors TGFβ1, VEGFA and lactate, and potently inhibited T cell proliferation.

**Conclusion:**

Co-cultures of organoids and immortalized CAFs recapitulate the histological, biophysical, and immunosuppressive features of aggressive mesenchymal-like human CRC. The model can be used to study the mechanisms of immunosuppression and to test therapeutic strategies targeting the cross-talk between CAFs and cancer cells. It can be further modified to represent distinct colon cancer subtypes and (organ-specific) microenvironments.

## Introduction

Colon cancer is one of the leading causes of cancer-related mortality in adults (1). Histopathological, genetic, immunological and molecular analysis of colon cancer has yielded insight into the heterogeneous nature of the disease (2–7). Characteristics like microsatellite instability (MSI), the presence of activating mutations in *KRAS* or *BRAF*, and/or infiltration by T cells, show correlations with prognosis and/or treatment response. Some of this information has been used to devise more personalized strategies of (targeted) anti-cancer treatment (8, 9). Despite these advances, the 5-year survival for patients with metastatic colon cancer remains very poor (~15%) and has only marginally improved over the past decade (10). A notable exception are patients with metastatic tumors with high levels of MSI (MSI-H), representing ~3-5% of all cases of metastatic colon cancer. Treatment of MSI-H metastases with anti-Programmed Death-1 (PD-1) yields significantly better and more durable anti-tumor responses, better progression-free survival, with lower toxicity, when compared to standard chemotherapy (11, 12). Nevertheless, even after anti-PD-1 treatment, more than half of the patients experience disease progression within 5 years (12). In addition, only patients with MSI-H tumors benefit from immune checkpoint blockade, while MSI-low (MSI-L) tumors generally fail to respond (13, 14). Thus, insight into the mechanisms underlying immunotherapy failure may form the basis for designing rational combination treatment strategies (15).

The success of immunotherapy depends on the infiltration of tumors by immune cells and on signals from the tumor microenvironment (TME) that impact immune cell differentiation and function (16). The TME consists of extra-cellular matrix (ECM) components, endothelial cells, immune cells and cancer-associated fibroblasts (CAFs), and plays a major role in determining immune cell function in cancer (17). CAFs in particular promote colon cancer initiation (18, 19), progression (20), metastasis formation (21) and therapy resistance (22, 23). When activated by cancer cells, CAFs may in turn promote aggressive cancer cell behavior (24). In addition, CAFs contribute to the generation of an immunosuppressive TME (25–28). Functionally, the continuous deposition of extracellular matrix (ECM) components is a critical CAF-mediated feature impacting multiple aspects of aggressive tumor cell behavior and immunosuppression (29).

In colon cancer, the ‘mesenchymal-like’ consensus molecular subtype 4 (CMS4) is characterized by a high CAF content (2) and by a gene expression program reflecting angiogenesis, inflammation and immunosuppression (30). Despite the immunosuppressive nature of CMS4 MSI-L colon cancer, cancer-specific neo-antigen-reactive T cells have been isolated from such tumors (31). Therefore, boosting the activity of tumor-reactive T cells in CMS4 colon cancer may be a promising therapeutic strategy (31). Indeed, in a pre-clinical mouse model of stroma-rich MSI-L colon cancer, inhibition of immunosuppressive TGFβ signaling was sufficient to evoke a response to anti-PD1 therapy (32).

The design and testing of novel therapeutic strategies requires model systems for studying CMS4 (CAF-rich) colon cancer. Organoid technology has emerged as a superior culturing platform for modelling human colon cancer (33). Organoid culturing media are optimized for epithelial (tumor cell) growth. As a result, cells and matrix components from the TME are rapidly lost from such cultures, precluding the analysis of reciprocal tumor-cell/TME interactions. This is highly relevant for CMS4 colon cancer, in which the immunosuppressive TME is dominated by CAFs. Indeed, transcriptomic analyses of colon cancer organoid biobanks show that CMS4 is not well represented (34, 35). This is because the mesenchymal gene expression that defines CMS4 is largely derived from CAFs (36, 37), which are rapidly lost from organoid cultures. The precise roles that CAFs play in mediating aggressive CMS4 behavior therefore remain incompletely understood. To start unravelling how the interplay between tumor cells and CAFs determines tumor behavior, organoid-based model systems are required in which both cell types are co-cultured in a reproducible and robust manner.

Here, we present a long-term co-culture model of colon cancer organoids and CAFs in a highly standardized serum-free medium and an optimized ECM. Under these conditions, cancer cells and CAFs spontaneously organized into superstructures while stiffening and contracting the ECM. Epithelial glandular structures were in direct contact with CAF tracks, closely resembling the histology of colon tumors in patients. Single cell transcriptomic analyses further demonstrated the co-culture-induced generation of clinically relevant cancer cell and CAF phenotypes. Both cell types were required to generate an immunosuppressive milieu in which T cell proliferation was inhibited.

## Methods

### Human tissue

Patient’s tissue samples were obtained within the biobanking protocol METC 09-145. The protocol was approved by the Medical Ethical Committee of the Utrecht Medical Center (UMCU) in the Netherlands. Written informed consent was obtained from all patients.

### CAFs

For CAF isolation, colorectal liver metastasis were cut in to small pieces and digested using Liberase TH (Roche, 5401135001) for 30 min at 37 °C. The cell suspension was incubated in plastic dishes for 30 min. After 30 min the non-adhered cells were washed away and the adherent cells were incubated with CAF medium (**Table S1**). When the cells reached ~90% confluency they were detached from the plate using Trypsin and replated in a cell culture flask with a thin collagen coat (Corning, 354249) with CAF medium. Low-passage primary CAF cultures (typically P1-P3) were immortalized with lentiviral constructs encoding hTERT and BMI1. For this we used pLOX-CWBmi1 (Addgene #12240) and pLV-hTERT-IRES-hygro (Addgene #85140). Lentiviral production of these constructs was performed using a calcium phosphate transfection protocol in human embryonic kidney 293T (HEK293T) cells using the coding plasmid (15 µg), vsv-G (#12259, 7.5 µg) and psPAX2 (#12260, 7.5 µg). After 24 hours the medium was replaced by CAF medium. The next day the virus containing CAF medium, supplemented with Polybrene (Sigma-Aldrich, 8 µg/ml), was added to the CAFs for 24 hours at 37 °C. After 24 hours the virus containing medium was replaced with fresh CAF medium. Immortalized CAFs were passaged once a week using Trypsin. For the experiments passage numbers 10 through 20 were used. In this manuscript we made use of two CAF lines; LCAF5 and CR16. Both lines were generated from colorectal liver metastases. The primary tumors from LCAF5 and CR16 were located in the sigmoid colon and the ascending colon respectively. Their respective TNM classifications were pT3N0 and pT4N2.

### Cell titer glo

To check for differences in cell viability between the medium conditions we performed a CellTiter-Glo 3D (Promega, #G9681) assay according to the manufacturer’s instructions. In short, CAFs were grown at 80-90% confluency in 96-well plates. Culture plates were equilibrated to room temperature (RT) for 30 min after which 100µl of Cell-Titer-Glo reagent was added directly to the medium in the wells. The reagent lysed the cells and generated a luminescent signal proportional to the amount of ATP present. Luminescence was measured with the SpectraMax plate reader (Molecular Devices).

### Fibroblast activation protein flow cytometry

To check the mesenchymal nature of the immortalized CAFs we measured Fibroblast Activation Protein (FAP) expression using Flow Cytometry. CAFs were trypsinized, washed two times with FACS buffer (PBS/1% BSA/2.5 mM EDTA) and counted. CAFs were stained with either an anti-FAP antibody (PE-conjugated, R&D Systems, FAB-3715P, 10µl/106 cells) or isotype control (Thermo Fisher, 12-4714-82, 5µl/106 cells). After 30 min incubation on ice, cells were washed two times and fluorescence was measured with the LSRFortessa from Becton Dickinson (BD) and analyzed using FlowJo software version 10.8.1.

### Organoids

We made use of 3 organoid lines; CR16, CR39 and P19bT. The organoid lines CR16 and CR39 were generated from resection material of metachronous colorectal liver metastasis. Their corresponding primary tumors were located in the ascending colon. The TNM classifications were pT4N2M0 and pT3N0M0 respectively. P19bt was previously described (35), and originates from a primary tumor. The organoid CR16 and CAF line CR16CAF are paired, they originate from the same colorectal liver metastasis. The other organoids and CAFs are from different patients. Organoids were cultured by embedding in Matrigel (Corning, NY, USA) and were passaged once a week through dissociation with TrypLE Express (Gibco, 12604021) for 5-10 min at 37°C. All organoids were cultured in full organoid medium (**Table S1**).

To generate green fluorescent organoids we performed two consecutive lentiviral transductions. The plasmids pBOB-Lck-GFP (Addgene #118738) and H2BmNeon were used for plasma membrane GFP and nuclear mNeon respectively. Lentiviral production of these constructs was performed using the same calcium phosphate transfection protocol as described above. 24 hours after the transfection, the medium of the HEK293T cells was replaced by basal organoid medium (**Table S1**). The next day organoids were dissociated into single cells and the virus containing medium (supplemented with Polybrene (Sigma-Aldrich, 8 µg/ml), N-acetylcysteine (Sigma-Aldrich, 1.25 mM) and ROCK-inhibitor Y-27632 (Sigma-Aldrich, 10 μM) was added to the cells for 24 hours at 37 °C. After 24 hours the cells were washed 2 times in PBS and plated in Matrigel. After the first passage organoids were grown in the presence of bleomycin (Lck-GFP) and puromycin (H2BmNeon), after two weeks organoids were sorted based on their green fluorescence using FACS.

### Co-culture

To create the co-cultures, 4 day old organoids were harvested from their Matrigel droplets using dispase (1mg/ml). Organoids were washed, counted and resuspended in co-culture matrix. This co-culture matrix consisted of 1,25mg/ml Collagen-I (Corning, 354249) neutralized with neutralization buffer in a 4:1 vol/vol ratio (5X neutralization buffer = dH20|alpha MEM50mg/ml|2%NaHCO|0.1M HEPES|Ph7.5) and Matrigel (Corning). A total of 600, small organoids were plated in 20ul droplets in a 48 well plate. CAFs were trypsinized and stained with Cellbrite red (Biotium, 30023, 5ul dye/10^6 cells). After washing and counting, 28.000 stained CAFs were resuspended in 200ul co-culture medium (**Table S1**). For the organoid mono-culture control condition we plated 600 small organoids in co-culture matrix with ‘empty’ co-culture medium and for the CAF mono-culture condition we plated an empty co-culture matrix droplet and added 28.000 CAFs in co-culture medium. After 5 days 100ul fresh co-culture medium was added to the wells. After 8 days the co-cultures were formed and medium and/or cells were harvested for further experiments.

### Live cell imaging

For live cell imaging a co-culture droplet was imaged every hour using the Leica SP8 microscope during the course of 16 hours, going from day 2 to day 3. Fiji was used for image processing. Maximal projection was used for Z-positions 4 till 7.

### Single cell RNA sequencing

#### Generating single cell suspensions

In order to sort single cells from the co- and mono-culture conditions, single cell suspensions were made. In brief, on day 8 of the experiment droplets were scooped out of the well and resuspended in Liberase to dissolve the co-culture matrix. Cell clusters and organoids were digested into single cells through incubation and resuspension with TrypLE. To create a single cell suspension for the adherent (2D) CAFs, wells, from which the CAF matrix droplet had been removed, were incubated with Trypsin and cells were harvested from the well plate. Sytox blue (Thermo Fisher, S34857, 1:1000) was added to all single cell suspensions as a live/dead cell marker. Viable single cells were sorted in 384-well cell capture plates using the BD FACSAria II.

#### Plate processing

The cell capture plates were ordered from Single Cell Discoveries, a single-cell sequencing service provider based in the Netherlands. Each well of a cell capture plate contains a small 50 µl droplet of barcoded primers and 10 μl of mineral oil (Sigma M8410). After sorting, plates were immediately spun and placed on dry ice. Plates were stored at -80° C. Plates were shipped on dry ice to Single Cell Discoveries, where single-cell RNA sequencing was performed according to an adapted version of the SORT-seq protocol (38) with primers described in van den Brink et al., 2017 (39). Cells were heat-lysed at 65° C followed by cDNA synthesis. After second-strand cDNA synthesis, all the barcoded material from one plate was pooled into one library and amplified using *in vitro* transcription (IVT). Following amplification, library preparation was done following the CEL-Seq2 protocol (40) to prepare a cDNA library for sequencing using TruSeq small RNA primers (Illumina). The DNA library was paired-end sequenced on an Illumina Nextseq™ 500, high output, with a 1x75 bp Illumina kit (read 1: 26 cycles, index read: 6 cycles, read 2: 60 cycles).

#### Data analysis

During sequencing, Read 1 was assigned 26 base pairs and was used to identify the Illumina library barcode, cell barcode, and UMI. Read 2 was assigned 60 base pairs and used to map to the reference transcriptome Homo sapiens (hg38) with BWA-MEM (41). Data was demultiplexed as described in Grün et al., 2014 (42). Briefly, Mapping and generation of count tables were automated using the MapAndGo script (https://github.com/anna-alemany/transcriptomics/tree/master/mapandgo). Downstream analysis was performed with Seurat v4.1.1 (43). Dimension reduction was performed with UMAP based on the first 30 principal components. Gene Ontology analysis was done using the package clusterProfiler on differential expression analysis (logFC > 0.5, min.pct = 0.25) of the Seurat clusters. To obtain DEGs that distinguished the organoid clusters, comparisons were made only between the organoid clusters. The same was done for the CAF clusters. Signatures were evaluated with the AddModuleScore function and retrieved from the Hallmark dataset of the molecular signature database (44) (9). New signatures were generated by performing differential expression analysis on the Seurat clusters (logFC > 0.75, min.pct = 0.5, min.diff.pct = 0.1).

### Analysis of publicly available single cell RNA seq data

Count matrices from Lee et al. (5) were extracted from the gene expression omnibus with identifiers GSE144735 and GSE132465. Data was processed with the Seurat single cell transform workflow (45). Next, stromal cells were extracted with a subset and the generated signatures, as well as the hallmark pathways, were evaluated with AddModuleScore function. The metadata, encompassing the CMS classification from the bulk RNA seq profiling, was extracted from Lee et al. and added to group stromal cells per cell subtype and CMS group.

### Immunofluorescence

Matrix droplets of all conditions (organoid mono-culture, CAF mono-culture and co-culture) were scooped out of the 48 wells and embedded in OCT. Matrix droplets were sectioned on a cryostat (7µm) and fixated with 4% paraformaldehyde (Sigma-Aldrich 158127) for 15 min at room RT. Sections were blocked for one hour with 5% normal goat serum and 0.3% Triton X-100 in PBS at RT. Primary antibodies rabbit anti-collagen IV (1:200, Abcam, ab19808), rabbit anti-LDHA (1:200, Cell Signaling, cs3582), rabbit anti-GAPDH (1:100, Cell Signaling, cs5174) were used overnight at 4C and detected using a secondary Goat anti Rabbit AlexaFluor 568 (1:600, Thermo Fisher, A11036) for one hour at RT. Nuclei were stained with DAPI. Sections were mounted with Prolong Gold antifade reagent and images were taken on the Zeiss LSM880 Confocal Laser Microscope. Immunofluorescence images were analyzed using QuPath v.0.3.0 (46). First the number of cells was quantified with the cell detection tool based on DAPI expression. Next the mean intensity of immunostaining per cell was quantified using the compute intensity function. Expression values were normalized to the mono-culture condition.

### Cell culture medium assays

#### Vascular endothelial growth factor alpha

The VEGF Quantikine ELISA kit (R&D Systems, DVE00) was used to detect VEGF-A levels in conditioned media from organoid mono-cultures, CAF mono-cultures and co-cultures. After 8 days, culture media from all three conditions was harvested and frozen at -80C until the assay. Samples were diluted 1:50 to 1:100 with fresh co-culture medium. Standards and samples were added to microplates pre-coated with a VEGF-A capture antibody. Samples were incubated for 2 hours at RT after which unbound substances were washed away. A substrate solution was added and color developed in proportion to the amount of VEGF-A present. Color development was stopped with a stop solution and the optical density of each well was determined with a microplate reader at 450nm. Standards and samples were assayed in duplicate.

#### Transforming growth factor bèta 1

To detect TGF-B1 levels in conditioned media from all co-culture conditions we used a Human TGF-B1 ELISA kit (Abcam, ab100647). Latent TGF-B1 was activated to the immunoreactive form by adding 1 N HCL into the cell culture supernatant and, after 10 minutes incubation, neutralizing the samples with 1.2 N NaOH/0.5 M HEPES. After activation, samples were diluted 1:5 with Assay Diluent from the kit. Standards and activated samples were added to microplates pre-coated with a TGF-B1 capture antibody. Bound TGF-B1 was detected using a biotinylated anti-TGF-B1 antibody, HRP-conjugated streptavidin and finally a substrate solution for color development proportional to the amount of TGF-B1. The intensity of the color was measured with a microplate reader at 450 nm. Standards and samples were assayed in duplicate.

#### Lactate

For the detection of lactate in conditioned media we used an enzymatic assay (Sigma-Aldrich, MAK064) according to the manufacturer’s instructions. Conditioned media were diluted 1:4 with fresh co-culture medium, 1ul of the medium was used for the assay. Standards and samples were added to a clear 96-well plate. The assay buffer, enzyme mix and probe were added to the wells and incubated for 30 min at RT. The absorbance was measured at 570nm with a microplate reader. Standards and samples were assayed in duplicate.

### T cell proliferation assay

To test the immunosuppressive effect of the conditioned media from the mono-culture and co-culture conditions we used a T cell proliferation assay. Cord blood T cells were harvested through Ficoll-paque separation of Cord Blood Mononuclear Cells (CBMCs) and subsequent CD3 MACS isolation. T cells were stained with CellTraceTM Violet (CTV) (Thermo Fisher, C3457) for 7 minutes at 37C. Cells were washed, counted and 100.000 T cells were plated per 96 well. To stimulate the T cells CD3/CD28 Dynabeads (Thermo Fisher, 40203D) were added in a ratio of 1 bead to 3 T cells, to the medium of all conditions except for the negative control. As a positive control, T cells received T cell medium (**Table S1**). The negative control condition contained T cell medium without Dynabeads and the medium control condition contained fresh co-culture medium to control for a possible effect of the co-culture medium on the T cells. The organoid, CAF and co-culture condition contained conditioned medium from the P19bt organoid mono-culture, the LCAF5 CAF mono-culture and the P19bt + LCAF5 co-culture condition respectively. All medium conditions were supplemented with 5% human serum (**Table S1**). After 4 days the T cells were harvested and CTV was measured using the Fortessa flowcytometer from Becton Dickinson (BD). Data analysis was performed using Flowjo software version 10.8.1.

### Statistical analysis

All statistical analyses were conducted in R version 4.0.5 for Windows. Significant differences between more than 2 cell clusters were calculated using the Kruskal-Wallis test. One-way ANOVA was used to compare differences in cytokine concentrations between the mono- and co-culture conditions, Šidák correction was used for multiple comparisons.

## Results

### Establishment of immortalized colorectal cancer-associated fibroblasts

**Figure 1:**
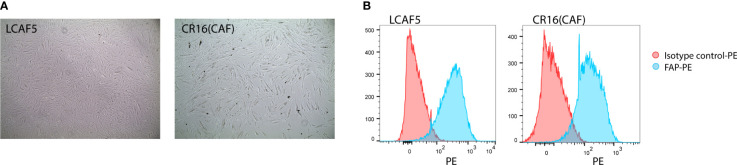
Establishment of immortalized colorectal cancer-associated fibroblasts. **(A)** Bright field images of two immortalized CAF cell lines, LCAF5 and CR16CAF, taken with the EVOS light microscope. **(B)** Histograms of FAP expression of both CAF cell lines measured using Flow Cytometry.

When freshly isolated from tumors, CAFs display a highly variable lifespan. Consequently, they cannot be used for the reproducible generation of long-term co-cultures. Therefore, we immortalized freshly isolated CAFs from colon cancer liver metastases by lentiviral expression of BMI and hTERT. This yielded 2 independent immortalized CAF cultures, LCAF5 and CR16CAF (**Figure 1A**), expressing high levels of the myofibroblast marker Fibroblast Activation Protein (FAP) (**Figure 1B**).

### Serum-free 3D culture conditions supporting long-term growth of patient-derived organoids and CAFs

Immortalized CAF cultures were established on cell culture plastics in medium containing 10% fetal calf serum (FCS). By contrast, organoids are cultured in a 3D basement membrane-like extracellular matrix (ECM) in medium with a defined growth factor composition that mimics the tissue of interest. Organoid growth media therefore lack undefined components like FCS. *Vice versa*, niche factors in organoid medium can inhibit fibroblast growth (**Figure S1**). To establish conditions that would support the long-term 3D culture of both cell types, we started with basal organoid medium (DMEM F12, HEPES, glutamax, penstrep) and added various components that are known to sustain organoid and fibroblast growth. In medium containing B27, fibroblast growth factor (FGF), insulin and platelet-derived growth factors A and B (PDGFA, PDGFB) the CAFs maintained similar proliferative capacity as in FCS-containing medium in which they were established (**Figure S1**). In addition, the stromal matrix protein collagen-I was added to the Matrigel basement membrane mix to improve CAF adhesion. These adjusted culturing conditions allowed long-term 3D growth of PDOs and CAFs together.

### Spontaneous reorganization of cancer cells and CAFs into macroscopic mini-tumors

**Figure 2:**
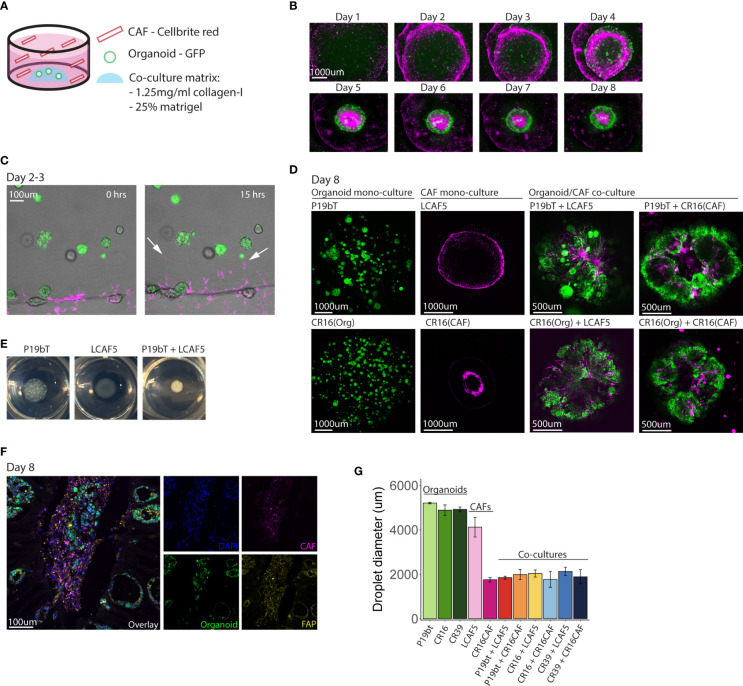
Spontaneous reorganization of cancer cells and CAFs into macroscopic mini-tumors. **(A)** Schematic overview of the co-culture model. Small GFP expressing organoids (n=600) are plated in a co-culture matrix containing Matrigel and collagen-I, CAFs stained with Cellbrite red dye (n=28,000) are plated in suspension in the well. **(B)** Fluorescence pictures taken with the EVOS M5000 microscope showing the time line of co-culture formation during the course of 8 days. The organoid P19bT is in green, LCAF5 is in magenta. **(C)** Two stills from a live cell imaging experiment conducted on the night of day 2 going on day 3 (full movie in **Supplemental Data**). The organoid is P19bT and the CAF line is LCAF5. During the course of 15 hours CAFs migrate into the matrix droplet, indicated by the white arrows. **(D)** Confocal images of organoid mono-cultures, CAF mono-cultures and Organoid/CAF co-cultures after 8 days. Organoids are in green, CAFs are in magenta **(E)** Pictures taken from the P19bT organoid mono-culture droplet, LCAF5 mono-culture droplet and P19bT/LCAF5 co-culture droplet on day 8. The co-culture droplet strongly contracted forming a macroscopically visible ‘mini tumor’. **(F)** Immunofluorescence image of FAP expression in a co-culture cryosection of P19bT organoid with LCAF5. **(G)** Diameter quantification of the matrix droplets after 8 days in culture. Bars represent the mean diameter of 5 droplets, error bars represent SD.

In order to distinguish cancer cells from CAFs in co-culture, cancer cells were transduced with a lentiviral vector encoding green fluorescent protein (GFP), while CAFs were ‘loaded’ with the dye Cellbrite Red. In the co-culture model, 600 small organoids were first plated in a co-culture matrix that contained Matrigel and collagen-I. 28.000 CAFs were then added to the co-culture medium in suspension (**Figure 2A**). The CAFs organized into a continuous circle surrounding the organoid-containing matrix droplets (**Figure 2B**), from where they entered the droplet in the first 2-3 days (**Figure 2C**, **Supplemental Movie 1**). Within the droplet, the CAFs organized into stromal tracks, much like what is observed in tumor histology. Simultaneously, the organoids reorganized along those CAF tracks. Ultimately, all individual organoids fused into a single entity surrounding the CAF tracks (**Figure 2D**, **Figure S2**), forming macroscopically visible ‘mini tumors’ (**Figure 2E**). To better characterize the organization of organoids and CAFs, co-culture matrix droplets were embedded in freezing medium (OCT) to create cryosections for immunocytochemistry using anti-FAP (**Figure 2F**). This revealed the presence of clusters of tumor cells residing in fields of FAP-positive CAFs, similar to normal colon tumor histology. We noted that, during cellular reorganization in the co-cultures, the matrix droplets displayed a significant contraction, yielding highly rigid matrix droplets, indicative of ECM reorganization and stiffening, as is commonly observed in solid cancers (47). Matrix stiffening and contraction were not observed in organoid monocultures, and less so in CAF monocultures, indicating that both cell types were required to induce this cancer-specific biophysical change (**Figure 2G**). These data show that co-culturing CAFs with tumor cells under optimized conditions results in spontaneous ECM reorganization and assembly of superstructures resembling tumor tissue. It is unclear what is causing the cellular reorganization. Since contraction of the matrix is only observed in conditions with CAFs present, it is difficult to separate the effects caused by the presence of CAFs and the effects caused by the contracting matrix.

### Co-culturing increases the fraction of cancer cells with reduced proliferation and partial EMT

**Figure 3:**
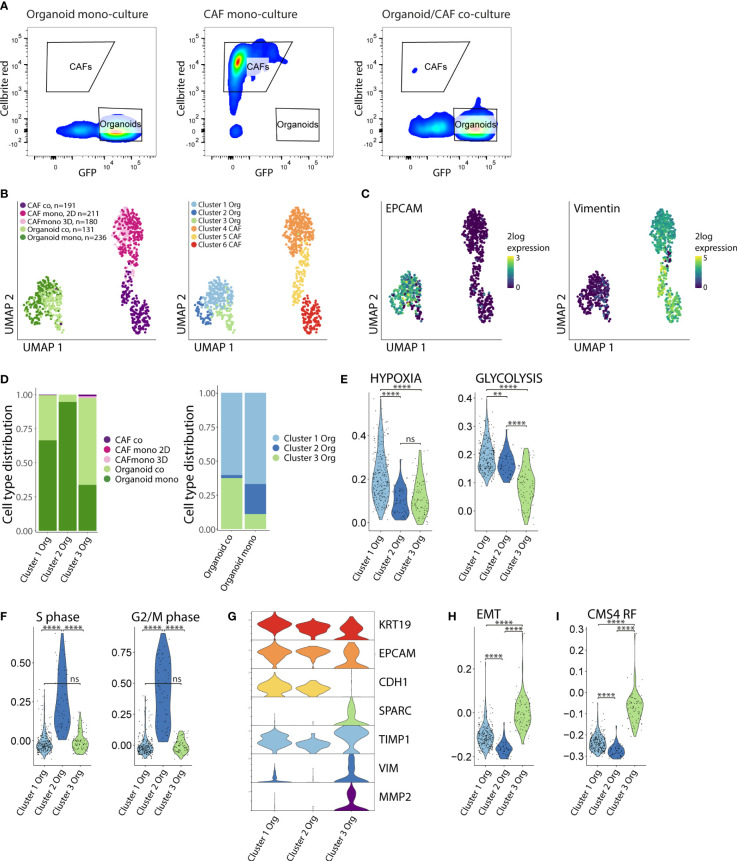
Co-culturing increases the fraction of cancer cells with reduced proliferation and partial EMT. **(A)** FACS sorting of organoids and CAFs from the mono- and co-culture conditions. Organoids were selected based on their GFP expression, CAFs were selected based on their Cellbrite red expression. Organoid = P19bT, CAF = LCAF5. **(B)** UMAP plots of single cell RNA seq data from organoids and CAFs from the mono- and co-culture conditions. CAF co = LCAF5 originating from the co-culture droplet, CAF mono 2D = LCAF 5 grown on the plastic of the CAF mono-culture condition, CAF 3D = LCAF5 grown on the matrix droplet of the CAF mono-culture condition, Organoid co = P19bT originating from the co-culture droplet, Organoid mono = P19bT originating from the mono-culture droplet. Graph based clustering analysis defined 3 organoid clusters and 3 CAF clusters. **(C)** Feature plot showing EPCAM and Vimentin expression. **(D)** Bar charts with cell type distribution of the 3 organoid clusters across the experimental conditions. **(E)** Violin plots showing the expression of Hypoxia and Glycolysis hallmark gene signatures across the 3 organoid clusters. **(F)** Violin plots showing the expression scores of S-phase marker genes (N=43) and G2/M-phase marker genes (N=54) across the 3 organoid clusters. Cluster 2 shows the highest expression of cell cycle genes. **(G)** Violin plots showing the expression of epithelial and mesenchymal genes across the 3 organoid clusters. Cluster 3 shows a lower expression of epithelial genes and a higher expression of mesenchymal genes compared to cluster 1 and 2. **(H)** Violin plot showing the expression of the Epithelial to Mesenchymal (EMT) Hallmark gene signature across the 3 organoid clusters. Cluster 3 cancer cells show the highest EMT gene signature scores. **(I)** Violin plot showing the module score of the CMS4 Random Forest (CMS4 RF) classifier genes (N=143). Cluster 3 shows the highest CMS4 RF signature score. ns, non-significant, Ns = p > 0.05, ** = p < 0.01,**** = p < 0.0001.

Next, we aimed to gain mechanistic insight into how CAFs and tumor cells would influence each other’s phenotype to induce ECM remodeling and cellular reorganization. To this end, we performed single cell RNA sequencing of both cell types isolated from mono-cultures (CAF-2D, CAF-3D, organoids) and co-cultures (CAF/organoids) by FACS sorting (**Figure 3A**, **Figure S3**). In total, we analyzed 949 cells. Dimensionality reduction and graph-based clustering analysis identified 3 cancer cell clusters (clusters 1-3) and 3 CAF clusters (clusters 4-6), marked by expression of EPCAM and Vimentin respectively (**Figures 3B, C**). Co-culturing of cancer cells with CAFs resulted in a decrease of cluster-2 cells while cluster-3 cells strongly expanded (**Figure 3D**). To gain insight into the phenotypic differences between the distinct clusters of cancer cells and CAFs we performed differential gene expression analysis. We classified the differentially expressed genes (DEG) into functional biological groups using gene ontology (GO) analysis (**Figure S4**, **Table S2**). Cluster-1 cancer cells were characterized by the expression of genes associated with hypoxia and glycolysis (**Figure 3E**). Cluster-2, consisting of 95% mono-culture cancer cells, displayed the highest expression of proliferation–associated genes (**Figure 3F**). Cluster-3 cancer cells, predominantly composed of cancer cells in co-culture, showed lower expression of epithelial genes (KRT19, EPCAM, CDH1) and higher expression of mesenchymal genes (SPARC, TIMP1, VIM, MMP2) and gene sets (Hallmark EMT) reflecting a (partial) epithelial-to-mesenchymal transition (EMT) (**Figures 3G, H**). Furthermore, the CMS4-identifying gene set (CMS4 RF), derived from the original random forest classifier (2) (which consists of genes mostly expressed by CAFs) was expressed at significantly higher levels in cancer cells from cluster 3 (p <10×^-16^) (**Figure 3I**).

Collectively, these analyses show that the interaction between cancer cells and CAFs in co-culture induces a phenotypic shift in the cancer cells, from an epithelial to a hybrid epithelial-mesenchymal state, as is observed in CMS4 colon cancer.

### CAFs in co-culture acquire a hypoxic, glycolytic and matrix-remodeling phenotype

**Figure 4:**
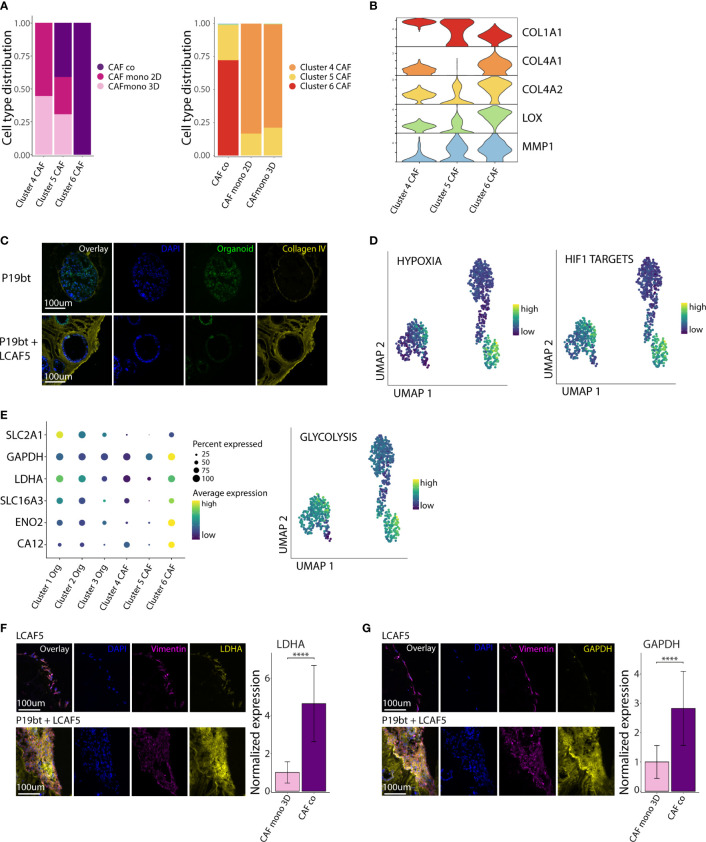
CAFs in co-culture acquire a hypoxic, glycolytic and matrix-remodeling phenotype. **(A)** Bar charts with cell type distribution of the 3 CAF clusters across the experimental conditions. **(B)** Stacked violin plot of genes related to Extra Cellular Matrix (ECM) remodeling across the 3 CAF clusters. **(C)** Immunofluorescence images of Collagen IV staining of the P19bT mono-culture and the P19bT + LCAF5 co-culture. **(D)** UMAP plots showing the expression of the Hypoxia hallmark gene signature and the HIF1 targets gene signature across all cells. **(E)** Gene expression dot plot showing the expression of several glycolysis associated genes and UMAP plot of the Glycolysis hallmark gene signature. **(F)** Immunofluorescence images of LDHA staining of the LCAF5 mono-culture and the P19bT + LCAF5 co-culture. Bar chart showing expression of LDHA in mono-cultured CAFs and co-cultured CAFs. Bars represent the mean normalized LDHA expression, error bars represent SD. **** = p <0.0001. **(G)** Immunofluorescence images of GAPDH staining of the LCAF5 mono-culture and the P19bT + LCAF5 co-culture. Bar chart showing expression of GAPDH in mono-cultured CAFs and co-cultured CAFs. Bars represent the mean normalized GAPDH expression, error bars represent SD. **** = p <0.0001.

We next analyzed the phenotypic differences between CAFs grown in mono-culture and in co-culture. Cluster 4 consisted entirely of CAFs isolated from mono-cultures (2D and 3D), while cluster 6 consisted entirely of CAFs isolated from co-cultures. Cluster 5 consisted of CAFs from both mono- and co-cultures (**Figure 4A**). All clusters expressed genes associated with ECM organization (**Figure S4**). Differential gene expression analysis showed major changes in the expression of various extracellular matrix genes, including downregulation of *COL1A1* (encoding stromal collagen-1) and upregulation of *COL4A1* and *COL4A2* (encoding basement membrane collagen-4) in the co-culture cluster 6 (**Figure 4B**). In addition, CAFs in co-culture expressed very high levels of the collagen-crosslinking enzyme *LOX* (lysyl oxidase) and the collagen-cleaving enzyme *MMP1* (matrix metalloproteinase-1). We used immunofluorescence analysis to visualize the matrix and assess its organization in co-cultures. This showed that CAF-produced collagen-4 formed a basement membrane-like meshwork interacting with cancer cells. (**Figure 4C**, **Figure S5**). Organization of the cancer cells along the collagen-4 basement membrane is accompanied by a partial restoration of apico-basal polarity, with the nuclei positioned close to the basal side. In addition, gene sets reflecting hypoxia (Hallmark) and hypoxia-inducible factor 1-alpha (HIF1A) targets (48) were significantly higher in CAFs in co-culture, when compared to those in mono-culture (p <10×^-16^) (**Figure 4D**). Further analysis revealed that co-culturing with cancer cells induced a major shift in CAF metabolism with increased expression of genes involved in glycolysis (*SLC2A, GAPDH, LDHA, SLC16A3, ENO2, CA12*), and the Hallmark gene set glycolysis (**Figure 4E**). We further examined the difference in CAF metabolism through immunofluorescence analysis. This showed a significantly higher expression of LDHA (**Figure 4F**) and GAPDH (**Figure 4G**) in co-cultured CAFs (p <0.0001).

### CAFs in co-culture resemble a subpopulation of CAFs enriched in CMS1 and CMS4 tumors

**Figure 5:**
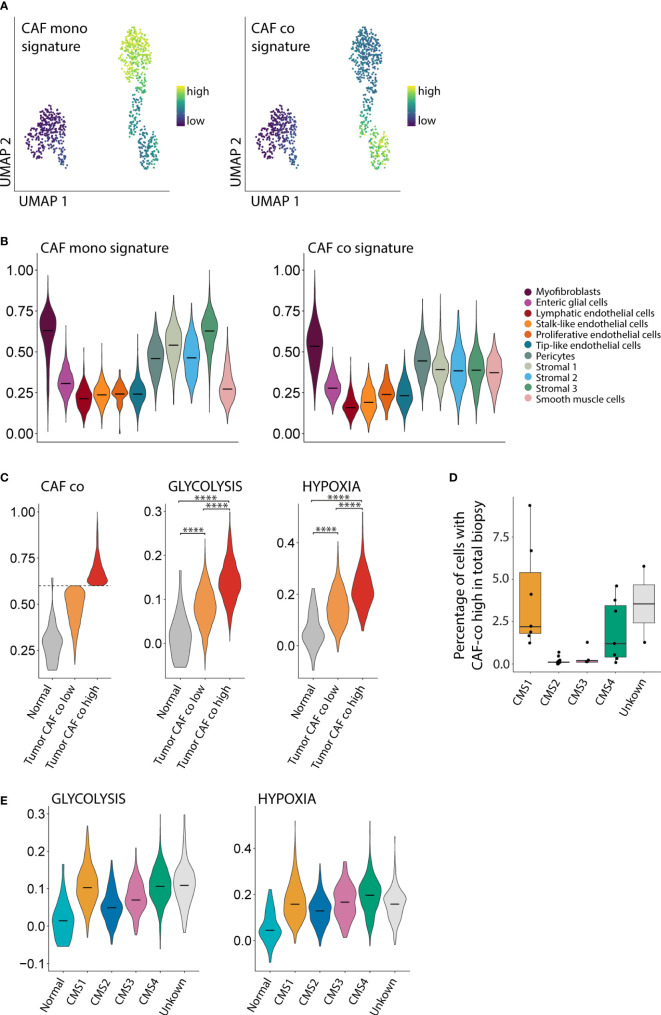
CAFs in co-culture resemble a subpopulation of CAFs enriched in CMS1 and CMS4 tumors. **(A)** UMAP plot showing the expression of mono- and co-culture CAF signatures. **(B)** Violin plot showing the expression of the mono- and co-culture CAF signature in stromal cells from a large single cell RNA seq dataset of primary colorectal cancer (N=29 patients, N = 13,583 cells). **(C)** Violin plot showing the expression of the CAF co-culture signature in myofibroblasts from normal tissue and in tumor myofibroblasts classified as CAF co low and CAF co high. **(D)** Boxplot showing the fraction of stromal cells classified as CAF co high within the total biopsy (epithelial, immune and stromal cells combined). **(E)** Violin plots showing the association between the Glycolysis and Hypoxia hallmark with the CMS1 and CMS4 molecular subtypes. **** = p <0.0001.

To study whether the CAF phenotypes in mono-culture and co-culture would resemble CAF phenotypes found in human colon cancer, we made use of a publicly available large single cell RNA seq dataset of primary colon tumors from 29 patients (5). First, we generated gene expression signatures for CAFs in mono-culture and CAFs in co-culture (**Figure 5A**, **Table S3**). The signature derived from the CAFs in mono-culture was highly expressed in various stromal cell types in human colon cancer, including myofibroblasts, pericytes, and stromal cell types 1-3 (**Figure 5B**). The signature derived from CAFs in co-culture was expressed in a more restricted fashion, mainly in myofibroblasts. There is some association between the CAF mono-culture and co-culture signatures and the endothelial cell subpopulations. This can be explained by the presence of many genes in both signatures that are associated with cell processes more than cell identity. To be completely certain that there is no endothelial cell contamination we checked the expression of PECAM1 (data not shown) which was absent from the CAFs as well as the organoids. Next, we analyzed the phenotypic differences of myofibroblasts isolated from human colon cancer specimens expressing either high or low levels of the *in vitro*-derived CAF co-culture signature. A subpopulation of human colon cancer myofibroblasts characterized by high expression of the co-culture signature, expressed significantly higher levels of the Hallmark glycolysis and hypoxia signatures than myofibroblasts with low expression of the CAF co-culture signature (p <10×^-16^) (**Figure 5C**). Next, we studied whether the subpopulation of hypoxic glycolytic CAFs in human colon cancer would be enriched in (a) particular molecular subtype of colon cancer. We found that fibroblasts expressing high levels of the CAF co-culture signature were mainly derived from CMS1 and CMS4 tumors (**Figure 5D**). In line with this result, CMS1 and CMS4-derived myofibroblasts expressed the highest levels of hypoxia and glycolysis signatures (**Figure 5E**). Together, the data indicate that the CAFs in co-culture resemble a subpopulation of CAFs with a hypoxic glycolytic phenotype that are enriched in human CMS1 and CMS4 colon cancer.

### Generation of an immunosuppressive microenvironment in co-cultures

**Figure 6:**
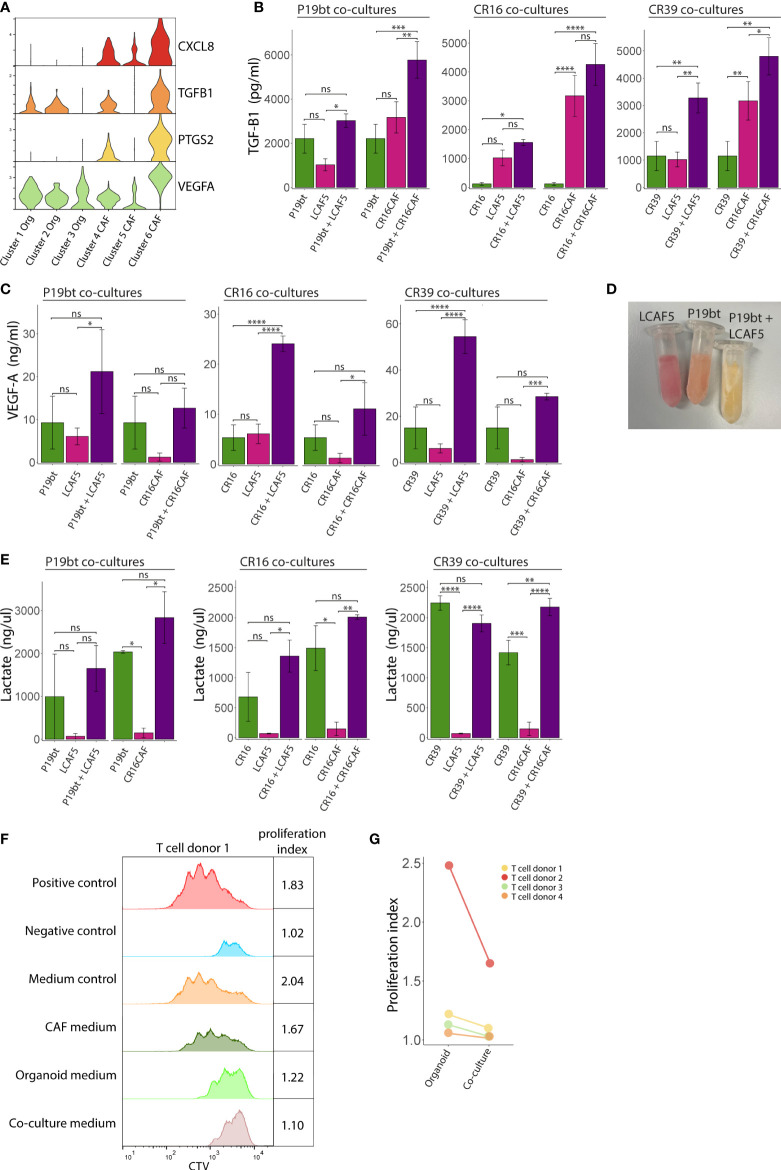
Generation of an immunosuppressive microenvironment in co-cultures. **(A)** Stacked Violin plots of immunosuppressive genes showing an increased expression by the co-cultured CAFs (Cluster 6). **(B)** Bar charts showing the TGF-B1 concentration in the medium from the mono- and co-culture conditions. Bars represent the mean concentration of 3 experiments, error bars represent SD. **(C)** Bar charts showing the VEGFA concentration in the medium from the mono- and co-culture conditions. Bars represent the mean concentration of 3 experiments, error bars represent SD. **(D)** Picture of Eppendorf tubes containing the conditioned medium of the CAF mono-culture condition LCAF5, the organoid mono-culture condition P19bT and the co-culture condition P19bT/LCAF5. The pH indicator in the medium clearly shows a decreased pH in the co-culture condition compared to the mono-culture conditions. **(E)** Bar charts showing the lactate concentration in the medium from the mono- and co-culture conditions, error bars represent SD. **(F)** Histogram showing the Cell Trace Violet (CTV) expression of one representative T cell proliferation experiment. The organoid conditioned medium peaks show a moderate inhibitory effect on T cell proliferation whereas the co-culture conditioned medium shows the strongest inhibition that somewhat resembles the peak of the negative control. **(G)** Dot plot showing T cell proliferation indices of the experimental conditions with organoid and co-culture conditioned medium, from four independent experiments. ns, non-significant, Ns = p > 0.05, * = p < 0.05, ** = p < 0.01, *** = p < 0.001, **** = p < 0.0001.

Several features of the co-cultures described above have previously been associated with immunosuppression in cancer, including a stiffening of the ECM (49, 50), hypoxia (51), and glycolytic metabolism (52, 53). Gene expression analysis showed that especially CAFs in co-culture (cluster-6) express very high levels of genes involved in immunosuppression, including *CXCL8* (interleukin-8 (IL8)), *PTGS2* (cyclooxygenase-2), *TGFB1* and *VEGFA* (**Figure 6A**). Analysis of the media conditioned by mono- or co-cultures showed that co-cultures generally secrete higher levels of TGFβ1 (**Figure 6B**), VEGFA (**Figure 6C**), and lactate (**Figure 6D, E**) than mono-cultures, indicating the generation of a potentially immunosuppressive milieu. We see differences in the amount of secreted factors between the co-cultures with different CAF lines e.g. CR16CAF secretes higher levels of TGFB1 compared to LCAF5, reflecting the heterogeneity of CAFs. To test the immunosuppressive potential directly we analyzed the effect of the conditioned media on the activation of T cells isolated from four distinct donors. Media isolated from CAF or cancer cell mono-cultures had no effect or a moderate inhibitory effect on T cell proliferation respectively. By contrast, media isolated from CAF/cancer cell co-cultures potently inhibited T cell proliferation in all four cases (**Figures 6F, G**, **S6, S7**, **Table S4**). These data show that part of the phenotype changes caused by co-culturing CAFs and cancer cells, lead to the generation of an immunosuppressive microenvironment.

## Discussion

Organoid technology has revolutionized cancer research in the past decade (54). However, organoid cultures are typically optimized for growth and expansion of epithelial (cancer) cells. Cells from the tumor microenvironment (including fibroblasts) are generally rapidly lost from organoid cultures. Therefore, the generation of advanced co-culture models in which non-epithelial cell types are represented and maintained is a major challenge in the organoid research field (55). In the current report we present a robust long-term co-culture model involving CRC organoids and stromal CAFs and show that multiple aspects of the immunosuppressive nature of stroma-rich cancers are recapitulated.

In previous reports CAFs were co-cultured with traditional colon cancer cell lines in collagen gels (56) or with colon cancer organoids in a transwell system, which prevents physical interactions between the cell types (57). Interestingly, in the former report (56), bulk gene expression profiling of 3-day old co-cultures showed that gene-sets reflecting hypoxia, ECM deposition, angiogenesis, and EMT were highly expressed in the co-cultures, very similar to what we find in the co-cultures described here. Additional reports confirm the induction of an EMT phenotype by CAF secreted factors *in vitro* (58). In our model we observed a phenotypic shift of cancer cells towards a partial EMT state upon co-culturing with CAFs. At the same time we observed a restoration of organoid polarity in the co-culture droplets. Intuitively these two phenomena seem to be in conflict with each other. However collective cell migration, or cluster migration, in which cell-cell junctions and polarity are retained in combination with the increase of mesenchymal markers to facilitate migration, shows a strong metastatic potential and is associated with partial EMT (59, 60). Robustness of the co-culture model is ensured by the immortalization of tumor-derived CAF cultures, allowing the generation of long-term and reproducible co-cultures. In addition, the composition of both the serum-free medium and the ECM is completely defined. Moreover, the system is highly versatile as it allows the use of existing living PDO biobanks to generate models for specific colon cancer subtypes, in co-culture with specific types of immortalized CAFs.

The induction of a tumor-suppressive microenvironment in the co-cultures was paralleled by a partial EMT in the tumor cells, implying that immunosuppression and aggressive cancer cell behavior are closely intertwined processes. Moreover, the reciprocal influence of cancer cells and CAFs is apparently sufficient to drive both hallmarks of aggressive CRC in the absence of other stromal and immune cell types. Much research has focused on CAF heterogeneity and the presence of multiple CAF subtypes in the tumor microenvironment. Two major CAF subtypes, first mentioned in the context of pancreatic ductal adenocarcinoma (PDAC), are the myofibroblastic CAF (myCAF) with a matrix remodelling and contractile phenotype and the immunomodulating CAF (iCAF) with an immunosuppressive phenotype (61). The CAFs in our co-culture model express matrix remodeling genes associated with the myCAF phenotype and besides show a very strong contractile phenotype. However, the co-cultured CAFs also start expressing more inflammatory cytokines such as IL-8 and TGF-B. From this we conclude that our co-cultured CAFs show features of both myCAF and iCAF, perhaps best resembling the myofibroblast subpopulation expressing genes enriched for inflammatory cytokines (MF2) as described in the paper by Lee et al. (5). The results also show that the co-culture CAFs represent a specific subpopulation of glycolytic myofibroblasts that is present in human CRC, and enriched in CMS1 and CMS4. Interestingly, ‘aerobic glycolysis’ by CAFs can contribute to lactate production, which inhibits T cell function within the TME (53), but can also be taken up by tumor cells as an energy fuel in a process called ‘the reverse Warburg effect’ (62, 63). It is unclear what exactly causes this shift in CAF metabolism in our model. One possible explanation would be the stiffening of the ECM caused by the strong contraction of the co-culture droplets. Stiffening of the ECM is known to induce changes in metabolism such as increased glycolysis and hypoxia (64–67). We believe that the contraction and matrix stiffening is a valuable part of the model since mesenchymal tumors are characterized by a stiff and desmoplastic phenotype *in vivo* that we can now recapitulate *in vitro*. Additional work is needed to assess how cancer cells and CAFs change their energy metabolism upon co-culture, how these changes are coupled, and how they influence T cell infiltration and function. Proteogenomic analysis of colon cancer has revealed that glycolytic activity is negatively correlated with CD8 T cell infiltration, specifically in MSI-H cancers (4), making up the vast majority of CMS1 (2). This suggests that future studies on immunosuppression using colon cancer co-culture models should take the molecular subtypes into account as a relevant variable. The large ‘living biobanks’ of colon cancer-derived organoids (34, 35) provide ideal resources for starting to address this.

Although the co-culture system presented here faithfully recapitulates multiple aspects of the immunosuppressive TME in stroma-rich colon cancer, it does not capture the full complexity of the TME in growing tumors and metastases. For instance, tumor-associated macrophages, myeloid-derived suppressor cells and tumor-associated neutrophils may all contribute to immunosuppression within the TME (68), but are not represented in the current co-culture system. Furthermore, much of what we know about the immune contexture in cancer comes from the analysis of primary tumors, while systemic (immuno-) therapies target metastases growing at distant sites. The specific organ context is likely to have major impact on the immune microenvironment of growing distant metastases. For instance, bone metastases in preclinical models of prostate cancer are refractory to immune checkpoint blockade due to high levels of TGFβ in the bone microenvironment blocking T_H_1 differentiation (69). In addition, regulatory T cells play an important role in establishing immune tolerance in metastases growing in the liver, and this (local) phenomenon also suppresses systemic anti-tumor immunity (70). Likewise, the presence of peritoneal metastases and the formation of ascites is associated with a poor response to immune checkpoint blockade in CRC (71, 72).

In general, the influence of specific organ microenvironments on shaping the immune contexture of distant metastases and their response to immune checkpoint blockade is an important theme in immuno-oncology research (73, 74). The co-culture model system that we have developed in the current report forms a starting point for addressing this issue empirically. By rationally adding specific cell types, and/or cytokines and/or extracellular matrix components, organ-specific microenvironments can now start to be modelled to assess their effect on T cell behavior. Advanced co-culture systems combined with advanced technologies for analyzing tumor cell killing by T cells (75) will be instrumental in defining the mechanisms causing immunosuppression in a cancer subtype- and organ site-specific manner. Ultimately, this will guide the development of novel informed combination treatment strategies involving immunotherapies.

## Data availability statement

Data is deposited in GEO under the accession GSE214569.

## Ethics statement

The studies involving human participants were reviewed and approved by Medical Ethical Committee of the Utrecht Medical Center. The patients/participants provided their written informed consent to participate in this study.

## Author contributions

EW, LW, SV and JL generated the immortalized CAF lines. ES, EW, ED, LW, IA, AS, NP, JH, MMCV and SV performed the experiments. ES, MV, AS, SE and OK analyzed and interpreted the data. ES, MV and OK drafted the manuscript. RF, JR, SN, HS, MG, IB, SE and OK supervised the study and revised the manuscript. All authors contributed to the article and approved the submitted version.
