# Supplementary material for: Co-cultures of colon cancer cells and cancer-associated fibroblasts recapitulate the aggressive features of mesenchymal-like colon cancer
Source: Front Immunol. 2023 May 16;14:1053920. doi: 10.3389/fimmu.2023.1053920 (PMC10228738; doi:10.3389/fimmu.2023.1053920)
Supplement: Supplementary file 1 [file DataSheet_1.pdf]

Table S1.

Medium type	Component	Source (catalog number)	Concentration
**Basal organoid medium**	Advanced-DMEM /F12	Gibco (12634-010)	1x
	HEPES buffer	Lonza (17737E)	10 mM
	Penicillin/Streptomycin	Gibco (15070-063)	50 U/ml
	GlutaMAX	Gibco (35050-038)	2 mM
**Full organoid medium**	Basal organoid medium		1x
	Noggin conditioned medium	293T-mNoggin-Fc cell line	100 ng/ml
	B27	Invitrogen (17504-044)	1x
	N-acetylcysteine (NAC)	Sigma-Aldrich (A9165)	1 mM
	A83-01	SignalChem (A09-900-05)	500 nM
	SB202190	Gentaur (A1632)	10 µM
**CAF medium**	DMEM high glucose	Gibco (D6429)	1x
	Penicillin/Streptomycin	Gibco (15070-063)	50 U/ml
	FCS	Bodinco BV, the Netherlands	10%
**Co-culture medium**	Basal organoid medium		1x
	B27	Invitrogen (17504-044)	1x
	FGF	ATCC (PCS-201-040)	5 ng/ml
	Insulin	ATCC (PCS-201-040)	5ug/ml
	PDGF-AB	Sigma Aldrich (P3326)	50ng/ml
**T cell medium**	RPMI 1640 with GlutaMAX and HEPES	Fisher Scientific (11554526)	1x
	Human serum	the Dutch blood bank “Sanquin”, 10 pooled donors (E8683R00)	5%
	Penicillin/Streptomycin	Gibco (11548876)	1%
	Betamercaptoethanol	Gibco (11528926)	50uM

Table S1. Components and concentrations of the used culture media.

Supplemental figure 1.

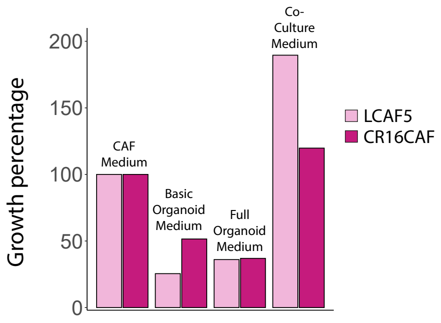

Figure S1. Cell Titer Glo assay showing the relative viability of LCAF5 and CR16CAF cultured in different media. The viability of the CAFs grown in CAF medium was set to 100%.

Supplemental figure 2.

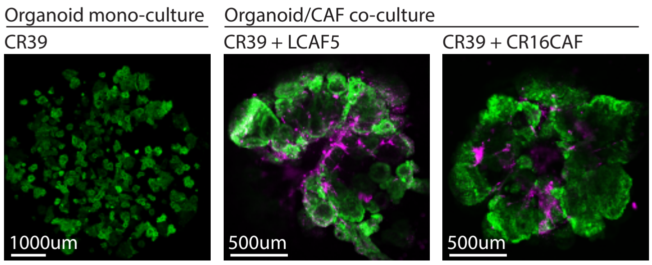

Figure S2. Confocal images of organoid mono-culture and Organoid/CAF co-cultures after 8 days. Organoids are in green, CAFs are in magenta

Supplemental figure 3.

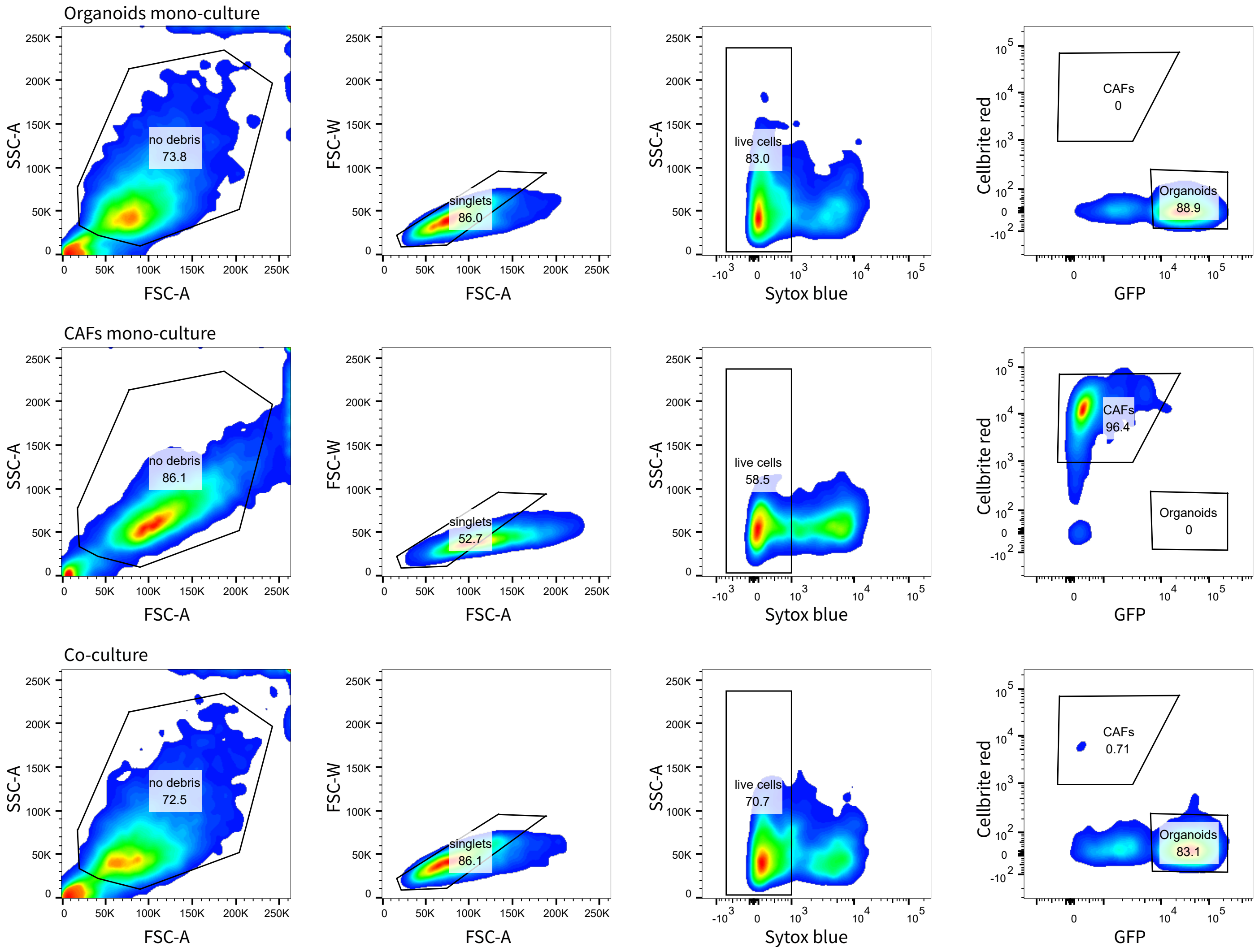

Figure S3. Gating strategy of the FACS sorting of organoids and CAFs from the mono- and co-culture conditions. In the first gate debris is gated out, in the second gate doublets are excluded and in the third gate live cells are selected. GFP positive organoids and Cellbrite red positive CAFs were sorted for single cell RNA sequencing.

Supplemental figure 4.

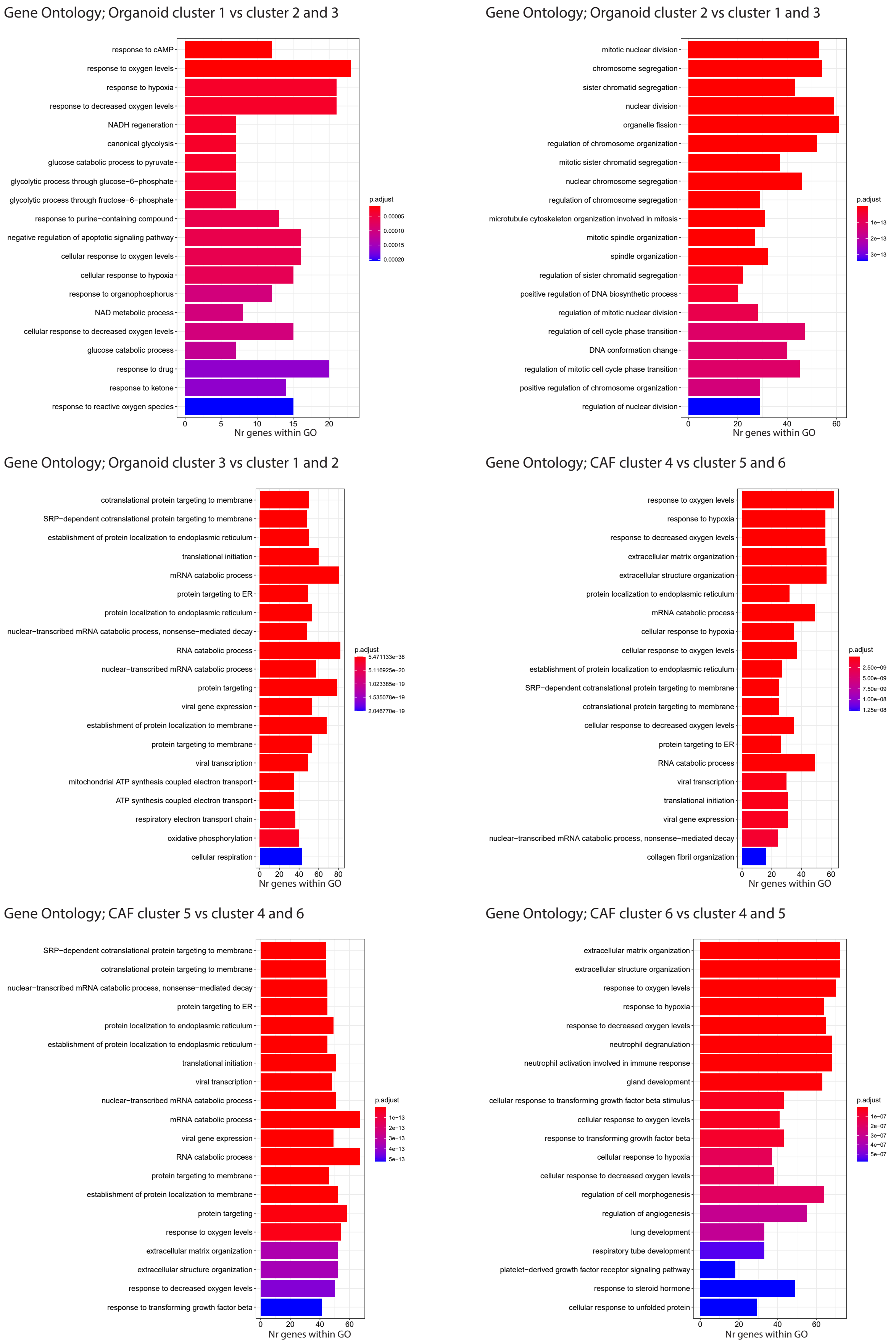

Figure S4. Gene Ontology analysis of differential gene expression analysis of the organoid and CAF clusters. The top 20 Gene Ontology categories per cluster are plotted. On the x-axis the number of expressed genes within a Gene Ontology category are shown, the color of the bar represents the adjusted p-value.

Supplemental figure 5.

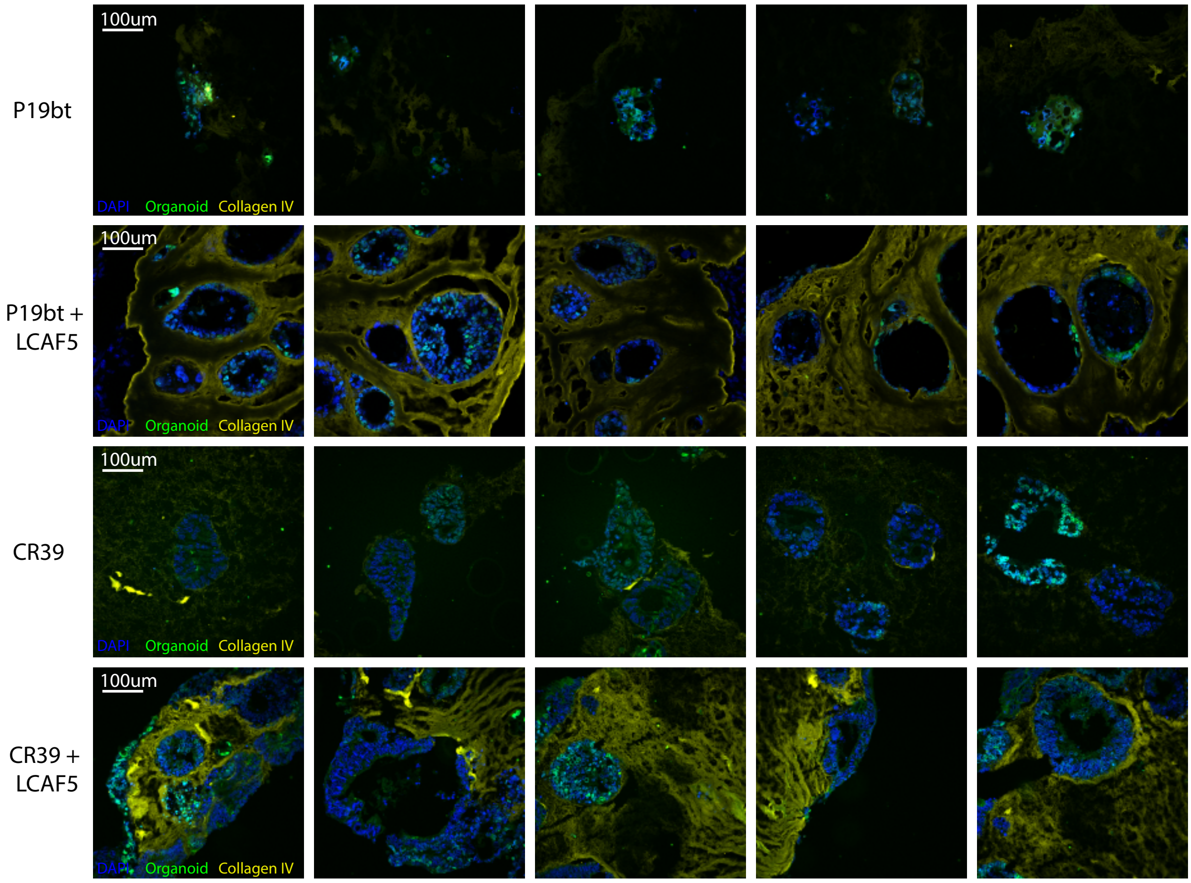

Figure S5. Immunofluorescence images of Collagen IV staining of the P19bT and CR39 mono-cultures and the P19bT/LCAF5 and CR39/LCAF5 co-cultures.

Supplemental figure 6.

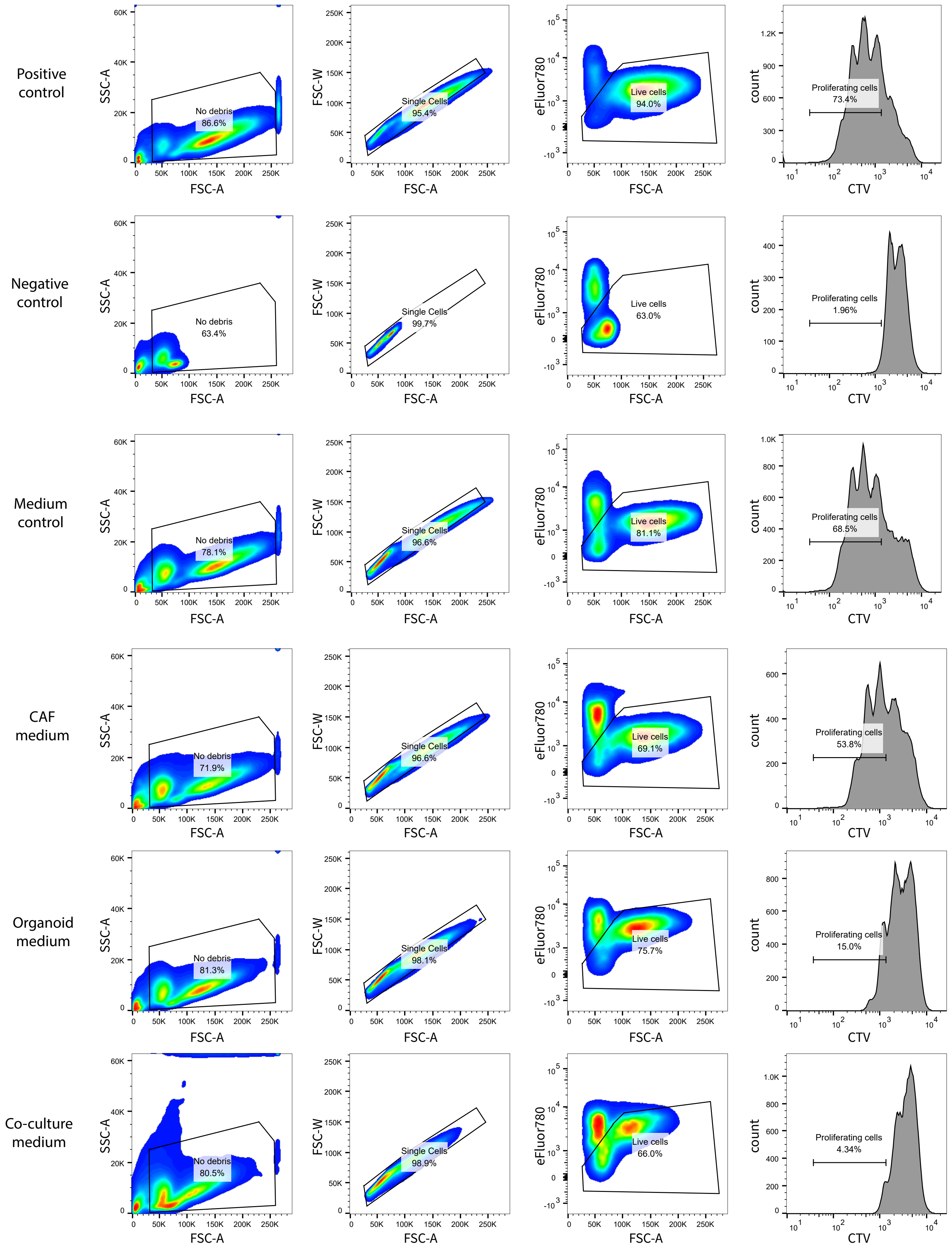

Figure S6. Gating strategy of T cells labeled with Cell Trace Violet (CTV) . In the first gate debris is gated out, in the second gate doublets are excluded and in the third gate live cells are selected. The histogram shows the percentage of proliferating T cells.

Supplemental figure 7.

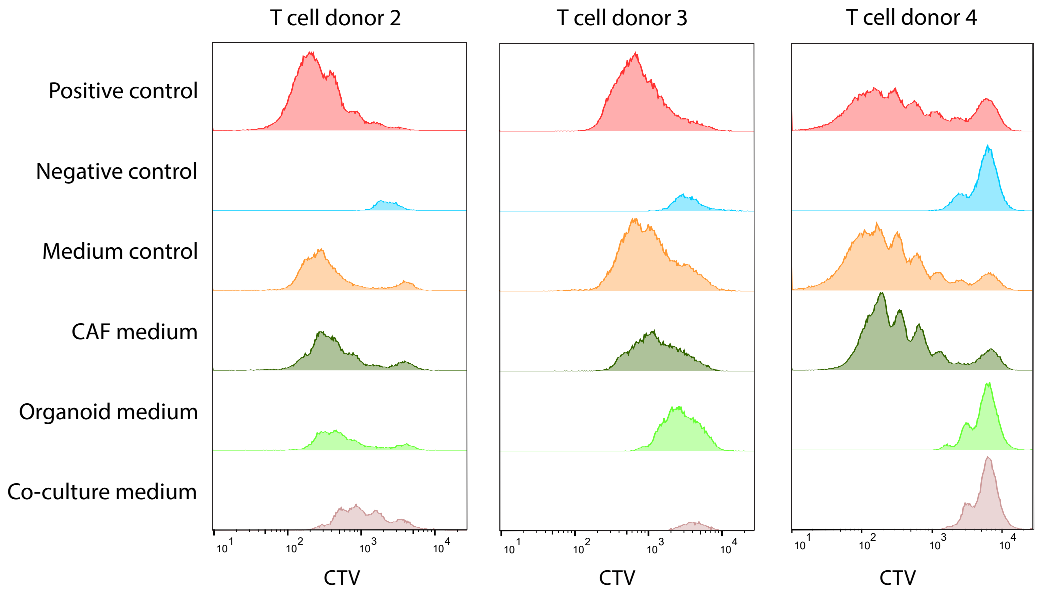

Figure S7. Histogram showing the Cell Trace Violet (CTV) expression of three T cell proliferation experiments with T cells from different donors. The number of peaks show the number of cell divisions. With each cell division CTV is divided over the two daughter cells, generating two cells with half the fluorescence. Peaks with low CTV fluorescence represent T cells that underwent multiple divisions.

Table S4.

**Condition**	**Number of live T cells per sample**	**Live cells: % of total events measured**
**Positive control**	103.406	78
**Negative control**	17.705	39
**Medium control**	66.360	59
**CAF medium**	44.432	48
**Organoid medium**	51.361	61
**Co-culture medium**	42.518	51

Table S4. Number of live T cells measured from the T cell proliferation assay with T cell donor 1.
